# Obituary

**Published:** 1910-01-01

**Authors:** 


### Obituary.

News has come by cable of the death of Mr. George Tyndale Lloyd at Mackay, Queensland. He took the degrees of M.B. and B.C. at Cambridge in 1889, studied at Bart's," and was medical officer of health at Mackay. He was in his 46th year.

We have to record the death of Lieut.-Col. Charles Egbert Wimond Bensley, late of the India Medical Service, from which he had retired. He took his M.D. at St. Andrews in 1856, gaining his membership of the Royal College of Surgeons, and his L.M. in the same year. He served in the Indian Mutiny in 1857-8 and gained the Mutiny Medal for his services.

Dr. William Eberhard, one of the oldest practitioners in Australia, died last month after a short illness. The deceased, who came from Braunschweig, in the Province of Hanover, was in his 88th year. In 1848 he arrived in South Australia with his brother, who predeceased him a few years ago. Dr. Eberhard resided at Tanunda during the whole of his time as a medical practitioner. He was appointed lodge surgeon about 1870, and held that position for over 25 years, to the satisfaction of all the brethren. He retired from his profession 15 years ago. Two years ago his wife died at the age of 92, shortly after the celebration of their golden wedding.

